# Supplementary figures and images for: Development and validation of a nomogram to predict the mortality risk in elderly patients with ARF
Source: PeerJ. 2021 Mar 9;9:e11016. doi: 10.7717/peerj.11016 (PMC7953875; doi:10.7717/peerj.11016)

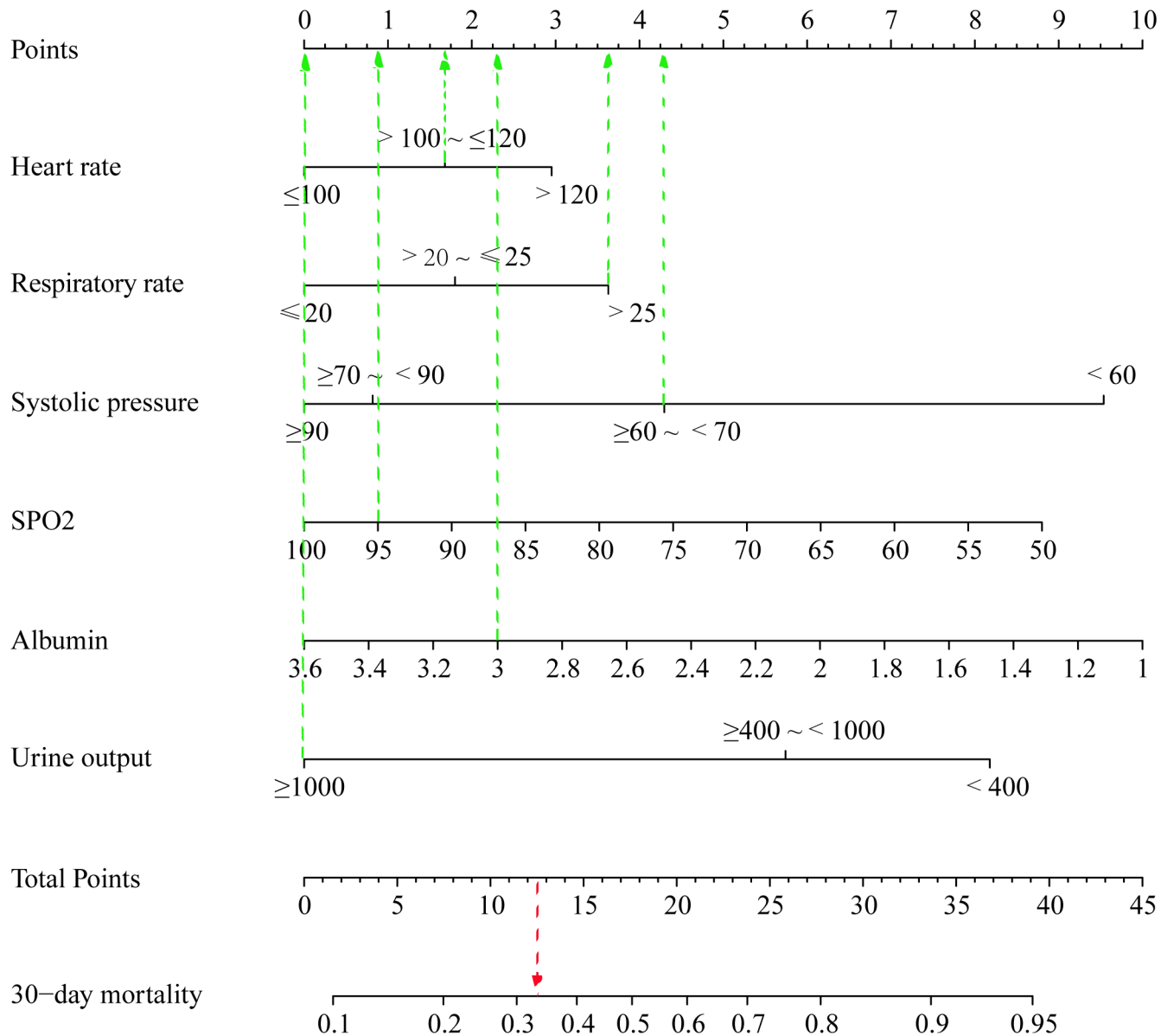

Supplement: Supplemental Information 3 — Example calculation: an elderly aute respiratory failure patient whoes heart rate was 110 bmp (1.5 points), respiratory rate was 26 per minute (3.5 points), systolic pressure 65 mmHg (4.2 points), SPO2 was 95% (0.9 point), albumin was 3 g/dL (2.2 points), 24 h urine output was 1200 ml (0 point). The total score was 12.3 points. This corresponds to 30-day mortality was 32%. [file peerj-09-11016-s003.pdf]

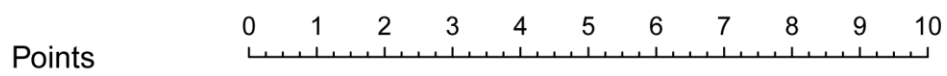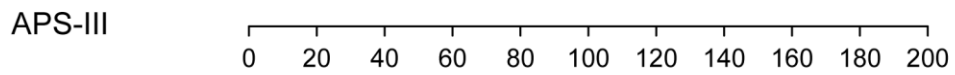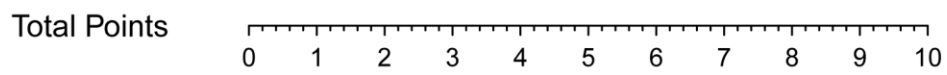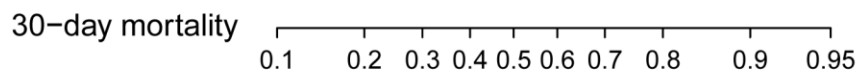

Supplement: Supplemental Information 4 [file peerj-09-11016-s004.pdf]

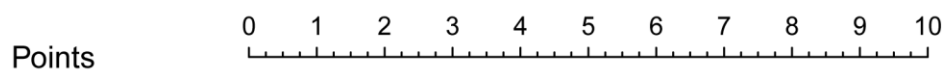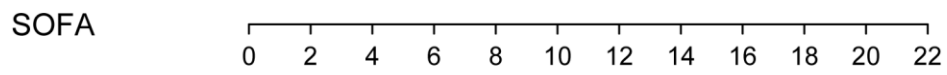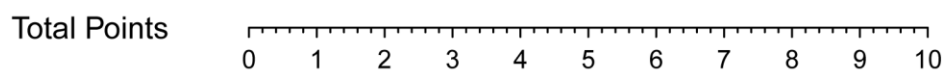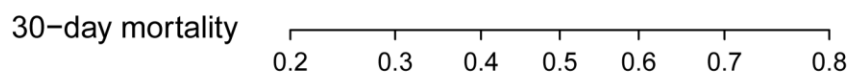

Supplement: Supplemental Information 5 [file peerj-09-11016-s005.pdf]
